# Accuracy of linear measurements obtained from stitched cone beam computed tomography images versus direct skull measurements

**DOI:** 10.12688/f1000research.17751.2

**Published:** 2020-03-02

**Authors:** Doaa Ahmed Fouad Hamed, Mostafa Mohamed El Dawlatly, Sahar Hosny El Dessouky, Reham Mohamed Hamdy

**Affiliations:** 1 Oral and Maxillofacial Radioalogy Department, Faculty of Dentistry, Cairo University, Cairo, Egypt; 2 Orthodontic Department, Cairo University, Cairo, Egypt

**Keywords:** Cone beam CT, linear measurements, accuracy, direct measurements, field of view, stitched images.

## Abstract

**Background**: To assess whether the linear measurements obtained from stitched cone beam computed tomography (CBCT) images were as accurate as the direct skull measurements.

**Methods**: Nine dry human skulls were marked with gutta-percha at reference points to obtain Twenty-two linear measurements on each skull. Ten measurements in the cranio-caudal plane, two measurements in the antero-posterior plane, and ten measurements in the medio-lateral plane. CBCT linear measurements obtained using stitching software were measured and compared with direct skull measurements.

**Results**: The absolute Dahlberg error between direct linear measurements and linear measurements on stitched CBCT images ranged from (0.07 mm to 0.41 mm). The relative Dahlberg error ranged from (0.2% to 1.8%). Moreover, Intra-class Correlation Coefficient (ICC) ranged from (0.97 to 1.0) indicating excellent agreement.

**Conclusion: **Stitched CBCT linear measurements were highly comparable to the direct skull measurements using a digital caliper.

## Introduction

The use of cone beam computed tomography (CBCT) machines in dentistry started in the second half of the 1990s ^1^. Now, CBCT is extensively used in the dental field for implant planning, in endodontics, maxillofacial surgeries and orthodontics ^2^.

In the field of orthodontics, analysis of cephalometric radiographs requires accurate identification of specific landmarks for precise measurements between these landmarks ^3^. As a consequence, the small field of view (FOV) CBCT systems available in small clinics cannot yet satisfy the needs of maxillofacial surgeons or orthodontists ^4^. Thus, visualizing all of these landmarks on the same scan is not always possible ^5^.

In order to compensate for this shortcoming, small FOV images can be scanned and then fused together to produce a single large FOV image. However, there are few data to show whether this fused image is as precise as a single image of the whole area of interest ^4, 6^.

Therefore, the aim of the current study was to assess the diagnostic accuracy of stitched CBCT linear measurements versus direct measurements on skulls.

## Methods

**Figure 1.**
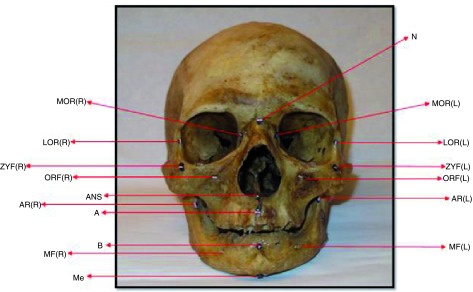
A photograph showing the frontal aspect of the skull.

The current study was conducted on nine dry human skulls obtained from the Anatomy department, Faculty of Medicine, Cairo University to avoid the exposure of living humans to unnecessary radiation doses. 26 anatomical landmarks were identified on each skull ( Table 1). Gutta percha cones (GE16121542, META BIOMED) were glued and used as radiopaque markers ( Figure 1– Figure 3).

**Table 1.** Showing the twenty-six anatomical landmarks identified on each skull.

Nasion (N)	The most anterior median point on the fronto-nasal suture.
Anterior nasal spine (ANS)	The most anterior median point (tip) of the anterior nasal spine of the maxilla.
Posterior nasal spine (PNS)	The most posterior median point (tip) of the posterior nasal spine of the maxilla.
A -point (A)	The point of maximum concavity in the midline of the alveolar process of the maxilla.
B-point (B)	The point of maximum concavity in the midline of the alveolar process of the mandible.
Menton (Me)	The most inferior midpoint of the chin on the outline of the mandibular symphysis.
Zygomatic foramen (ZYF) R&L	A small aperture on the convexity of the malar surface of the zygomatic bone near its center.
Condyle (Co) R&L	The most superior median point of the right and left condylar head.
Mandibular gonion (Go) R&L	Most posterior and inferior point of the curve between the body and ascending ramus on the right and left sides of the mandible.
Medial orbital wall (MOR) R&L	Point on the middle of the medial wall of the right and left orbits.
Lateral orbital wall (LOR) R&L	Point on the middle of the lateral wall of the right and left orbits.
Infra-orbital foramen (ORF) R&L	Foramen located below the infra-orbital margin of the right and left orbits.
Greater palatine foramen (GP) R&L	An aperture on the right and left postero-lateral aspects of the hard palate.
Mental foramen (MF) R&L	An aperture on the buccal surface of the mandible in the area of the mandibular premolars teeth on the right and left sides.
Anterior ramus (AR) R &L	Point on the middle of the anterior border of the right and left ramus.
Posterior ramus (PR) R&L	Point on the middle of the posterior border of the right and left ramus.

**Figure 2.**
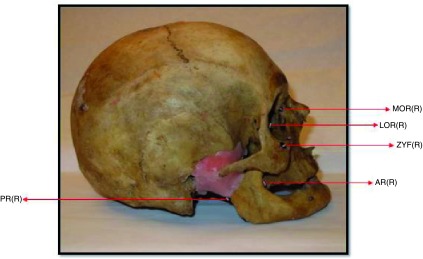
A photograph showing the lateral aspect of the skull.

**Figure 3.**
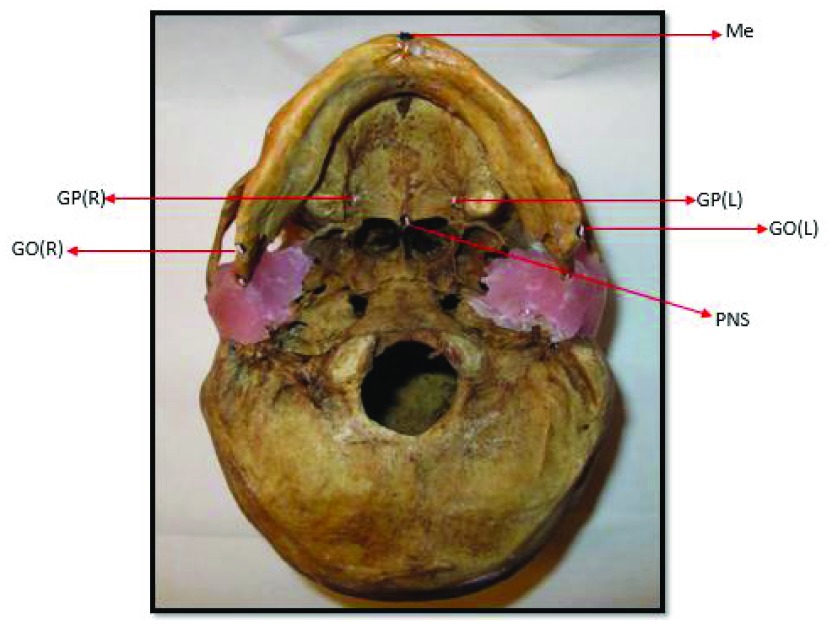
A photograph showing the skull base.

**Figure 4.**
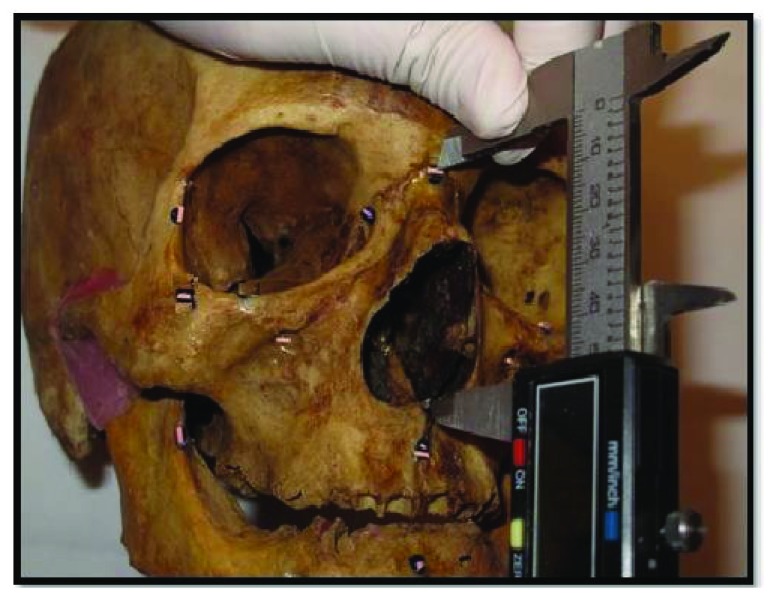
A photograph showing direct linear measurement from Nasion to Anterior nasal spine.

22 linear measurements were taken and recorded using a high precision sliding digital caliper (6400192, Allendale Electronics Ltd, Hertfordshire, UK). Ten measurements in the cranio-caudal plane ( Table 2), two measurements in the antero-posterior plane ( Table 3), and ten measurements in the medio-lateral plane ( Table 4). 22 direct linear measurements were measured and were considered to be the gold standard in the study ( Figure 4– Figure 6).

**Table 2.** Showing cranio-caudal linear measurements.

N-ANS	Nasion to anterior nasal spine.
N-A	Nasion to A-point.
N-B	Nasion to B-point.
N-Me	Nasion to menton.
ANS-A	Anterior nasal spine to A-point.
ANS-Me	Anterior nasal spine to menton.
B-Me	B-point to menton.
ORF(R)-MF(R)	Right infra-orbital foramen to right mental foramen.
ZYF(R)-MF(R)	Right zygomatic foramen to right mental foramen.
CO(R)-GO(R)	Right condyle to right gonion.

**Table 3.** Showing antero-posterior linear measurements.

ANS-PNS	Anterior nasal spine to posterior nasal spine.
AR(R)-PR(R)	Right anterior ramus to right posterior ramus.

**Table 4.** Showing medio-lateral linear measurements.

CO(R)-CO(L)	Right condyle to left condyle.
GO(R)-GO(L)	Right gonion to left gonion.
GP(R)-GP(L)	Right greater palatine foramen to left greater palatine foramen.
ORF(R)-ORF(L)	Right infra-orbital foramen to left infra-orbital foramen.
ZYF(R)-ZYF(L)	Right zygomatic foramen to left zygomatic foramen.
MOR(R)-MOR(L)	Right medial orbital wall to left medial orbital wall.
LOR(R)-LOR(L)	Right lateral orbital wall to left lateral orbital wall.
MF(R)-MF(L)	Right mental foramen to left mental foramen.
AR(R)-AR(L)	Right anterior ramus to left anterior ramus.
PR(R)-PR(L)	Right posterior ramus to left posterior ramus.

R – right, L - left

**Figure 5.**
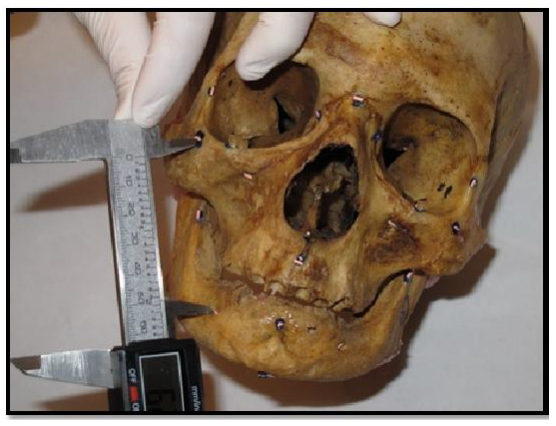
A photograph showing direct linear measurement from right Zygomatic foramen to right Mental foramen.

**Figure 6.**
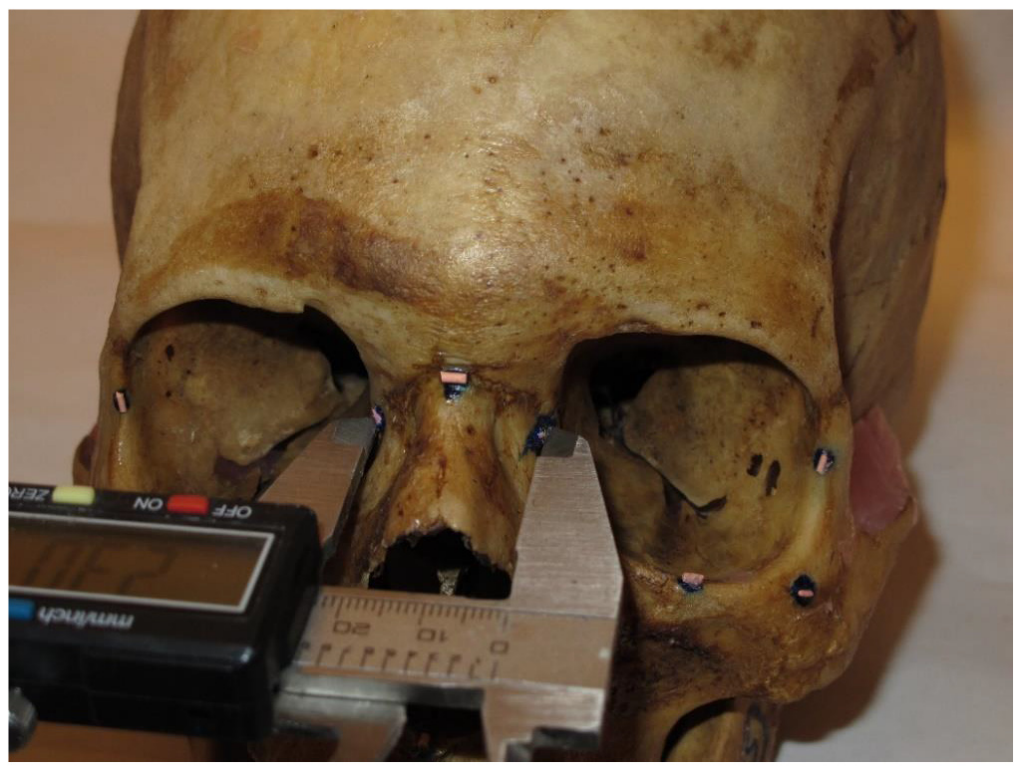
A photograph showing direct linear measurement from right Medial orbital wall to left Medial orbital wall.

For soft tissue simulation, the skulls were covered with 20 layers of pink modelling wax (1mm thick each) (Tenatex eco, Kemdent) to achieve an average of 14–16 mm wax thickness ^7^.

**Figure 7.**
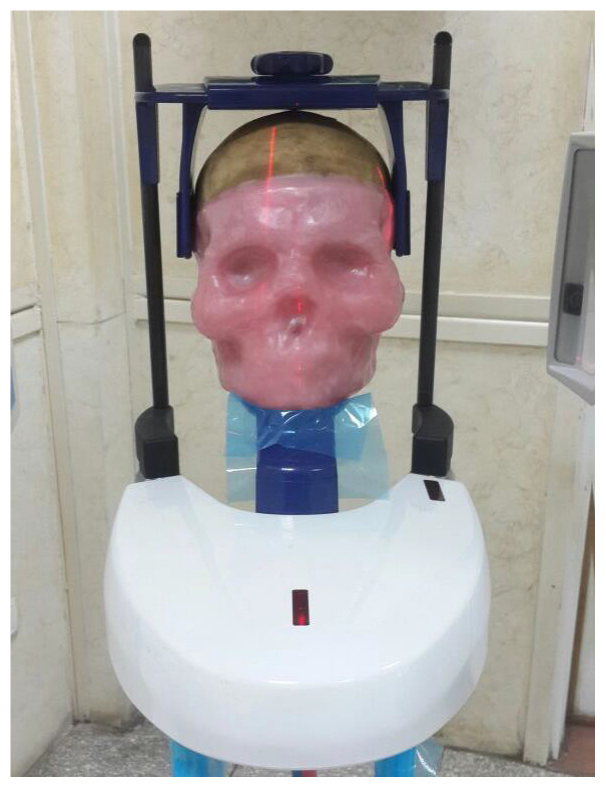
A photograph showing the skull centralized within the CBCT machine in the proper position.

**Figure 8.**
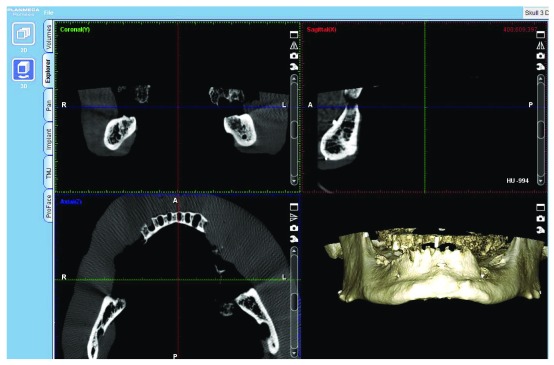
First field of view showing the mandible.

**Figure 9.**
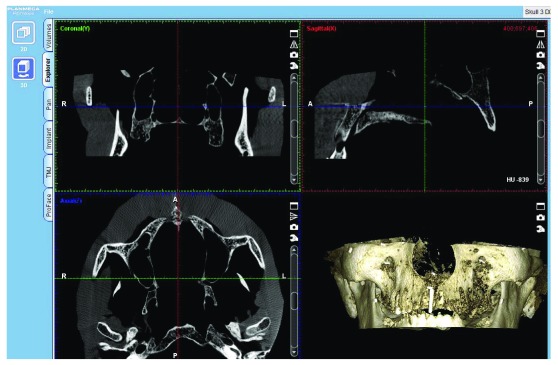
Second field of view showing the maxilla.

**Figure 10.**
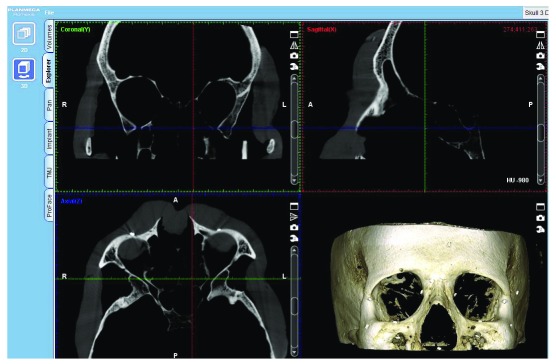
Third field of view showing the upper third of the face, orbits, frontal bone.

**Figure 11.**
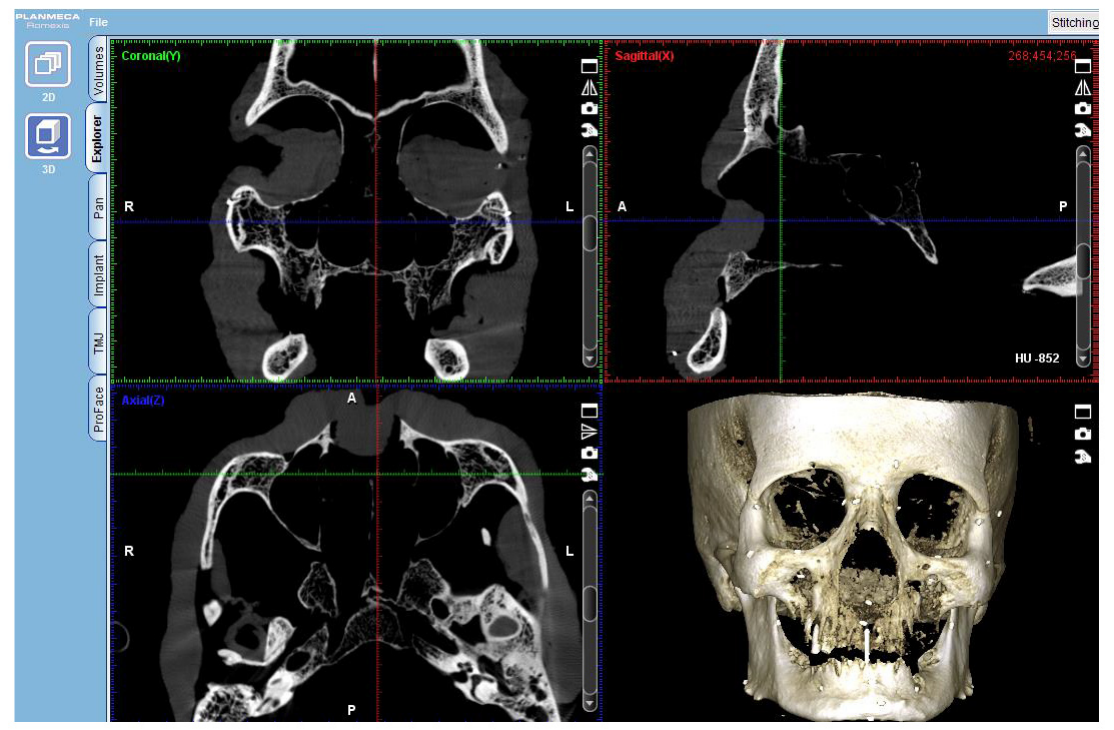
The final image showing the stitched three small fields of view into a single large one.

**Figure 12.**
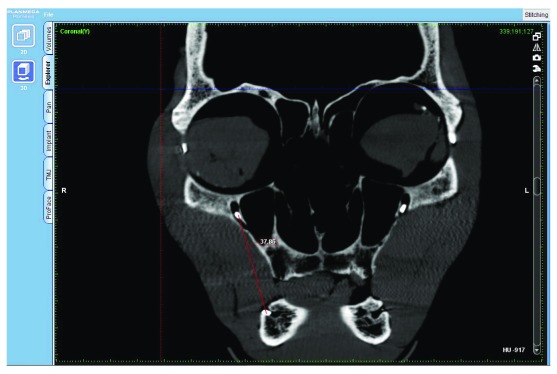
Coronal cut showing linear measurement of Orbital foramen (right)-Mental foramen (right) on a stitched image.

**Figure 13.**
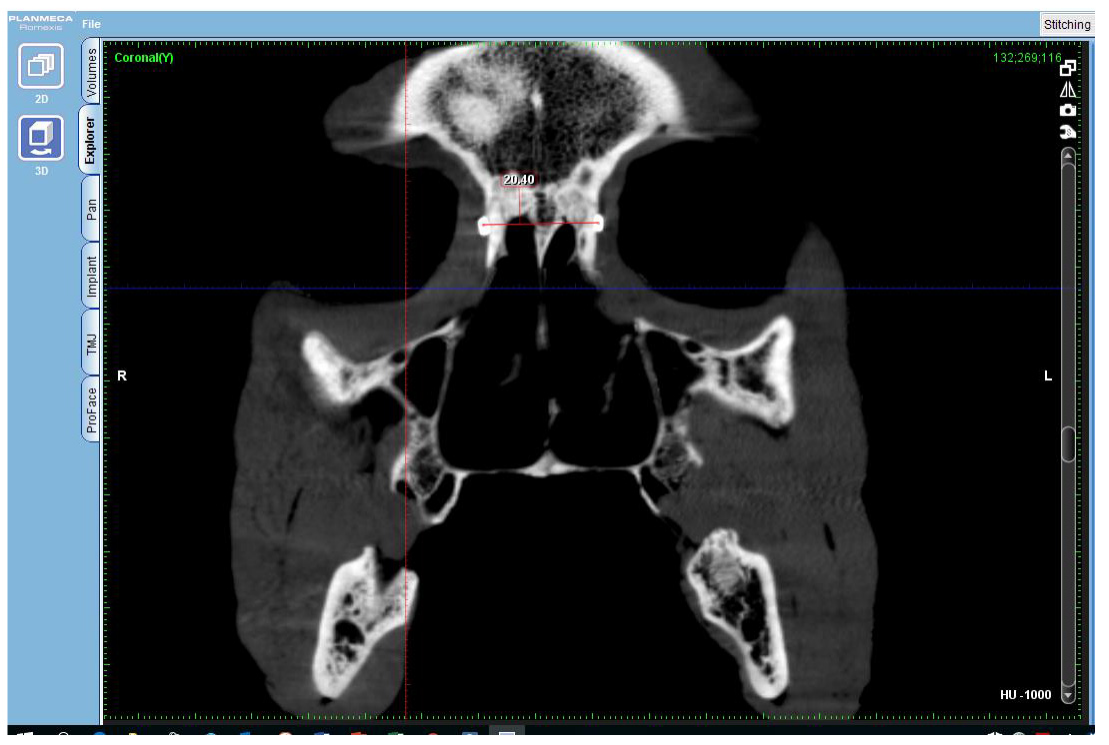
Coronal cut showing linear measurement of Medial orbital wall (right)-Medial orbital wall (left) on a stitched image.

The skulls were stabilized in the Planmeca ProMax 3D Mid CBCT machine using a wooden stand passing through the foramen magnum and were oriented using the laser beams ( Figure 7). Three consecutive FOVs were scanned: two scans each of the size 160 ×60 mm (single arch) for the mandible and the maxilla separately ( Figure 8 and Figure 9) and one scan with a FOV size 200 ×100 mm for the upper third of the face ( Figure 10). Each one of the three FOVs was scanned separately using a voxel resolution 0.2 mm, 90 kVp and 10 mA, then stitching was performed using Romexis software (Planmeca Romexis Viewer Launcher 4.5.0.R) creating one large volume ( Figure 11). After completion of the stitching procedure the linear measurements were obtained from the stitched CBCT images for a later comparison with the gold standard ( Figure 12 and Figure 13).

### Statistical analysis

Statistical analysis was performed on SPSS (version 17). For assessment of the agreement between all measurements with the reference method, Dahelberg error (DE), and Relative Dahelberg Error (RDE) were used together with Intra-class Correlation Coefficients (ICC) including the 95% confidence limits of the coefficient calculated assuming analysis of variance two-way mixed model ANOVA with absolute agreement on SPSS. To measure and quantify the size of the differences, Bland and Altman 95% confidence Limits of Agreements (LOA) were applied. 

## Results

### Error assessment of linear measurements conducted on stitched CBCT images versus direct skull measurements (the gold standard) ( Table 5)

The results of the current study showed that, the difference between the mean of the direct linear measurements and the mean of the linear measurements conducted on the stitched CBCT images ranged from (-0.25 mm to 0.5 mm), the positive and negative values indicating that there was no obvious pattern of over or underestimation in the stitched CBCT measurements.

**Table 5.** Comparing direct linear measurements and measurements conducted on stitched CBCT images.

								Bland & Altman Limits of Agreement (LOA)		Intra-class Correlation Coefficient		
Linear Measurements	Direct/ Stitched	Mean	SD	Dahlberg Error (DE)	Relative Dahlberg Error (RDE)	Mean of Difference (Reference - Stitched)	SD of the Difference	95%confidence limits			95%confidence limits	
								Lower	Upper	ICC	Lower	Upper
**N-A**	**Direct** **Reference**	56.71	4.35	0.21	0.4%	-0.11	0.30	-0.70	0.47	0.999	0.995	1.000
	**Stitched**	56.82	4.35									
**N-ANS**	**Direct** **Reference**	49.85	4.11	0.25	0.5%	-0.15	0.33	-0.81	0.50	0.998	0.992	1.000
	**Stitched**	50.00	4.20									
**N-Me**	**Direct** **Reference**	96.91	9.44	0.27	0.3%	0.21	0.33	-0.45	0.86	1.000	0.998	1.000
	**Stitched**	96.70	9.54									
**N-B**	**Direct** **Reference**	77.91	7.56	0.29	0.4%	-0.25	0.34	-0.92	0.42	0.999	0.995	1.000
	**Stitched**	78.16	7.51									
**ANS-A**	**Direct** **Reference**	7.28	1.62	0.09	1.3%	-0.09	0.10	-0.29	0.10	0.998	0.983	1.000
	**Stitched**	7.37	1.63									
**ANS-PNS**	**Direct** **Reference**	52.28	3.39	0.09	0.2%	0.02	0.14	-0.25	0.30	1.000	0.998	1.000
	**Stitched**	52.26	3.39									
**ANS-Me**	**Direct** **Reference**	46.95	6.52	0.23	0.5%	0.00	0.34	-0.66	0.67	0.999	0.997	1.000
	**Stitched**	46.95	6.59									
**B-Me**	**Direct** **Reference**	18.88	3.13	0.15	0.8%	0.18	0.12	-0.06	0.42	0.999	0.949	1.000
	**Stitched**	18.70	3.17									
**ORF(R)-ORF(L)**	**Direct** **Reference**	53.01	5.54	0.15	0.3%	-0.04	0.23	-0.48	0.41	1.000	0.998	1.000
	**Stitched**	53.04	5.43									
**MOR(R)-MOR(L)**	**Direct** **Reference**	22.94	1.89	0.41	1.8%	0.50	0.32	-0.13	1.12	0.974	0.318	0.996
	**Stitched**	22.45	1.67									
**LOR(R)-LOR(L)**	**Direct** **Reference**	96.81	3.86	0.22	0.2%	0.05	0.32	-0.58	0.68	0.998	0.993	1.000
	**Stitched**	96.76	3.88									
**ZYF(R)-ZYF(L)**	**Direct** **Reference**	96.95	6.18	0.17	0.2%	0.11	0.22	-0.33	0.55	1.000	0.998	1.000
	**Stitched**	96.85	6.26									
**GP(R)-GP(L)**	**Direct** **Reference**	31.60	2.69	0.15	0.5%	-0.07	0.21	-0.49	0.35	0.998	0.993	1.000
	**Stitched**	31.67	2.63									
**ORF(R)-MF(R)**	**Direct** **Reference**	46.61	5.08	0.22	0.5%	0.03	0.33	-0.62	0.68	0.999	0.996	1.000
	**Stitched**	46.59	5.14									
**ZYF(R)-MF(R)**	**Direct** **Reference**	57.56	6.70	0.15	0.3%	-0.06	0.22	-0.49	0.37	1.000	0.999	1.000
	**Stitched**	57.62	6.83									
**CO(R)-CO(L)**	**Direct** **Reference**	101.52	4.08	0.23	0.2%	-0.03	0.34	-0.69	0.63	0.998	0.993	1.000
	**Stitched**	101.55	3.91									
**GO(R)-GO(L)**	**Direct** **Reference**	94.19	8.06	0.27	0.3%	0.07	0.40	-0.72	0.86	0.999	0.998	1.000
	**Stitched**	94.12	8.11									
**CO(R)-GO(R)**	**Direct** **Reference**	58.52	6.65	0.13	0.2%	-0.01	0.20	-0.40	0.38	1.000	0.999	1.000
	**Stitched**	58.54	6.64									
**AR(R)-AR(L)**	**Direct** **Reference**	83.31	2.71	0.30	0.4%	0.04	0.45	-0.85	0.93	0.994	0.974	0.999
	**Stitched**	83.27	2.87									
**MF(R)- MF(L)**	**Direct** **Reference**	44.72	0.95	0.15	0.3%	-0.10	0.20	-0.49	0.29	0.987	0.945	0.997
	**Stitched**	44.82	0.95									
**PR(R)-PR(L)**	**Direct** **Reference**	96.32	5.30	0.38	0.4%	0.30	0.48	-0.64	1.24	0.997	0.986	0.999
	**Stitched**	96.02	5.14									
**AR(R)-PR(R)**	**Direct** **Reference**	33.07	2.48	0.07	0.2%	0.04	0.09	-0.13	0.21	1.000	0.999	1.000
	**Stitched**	33.03	2.48									

N – Nasion, ANS - Anterior nasal spine, PNS - Posterior nasal spine, A – A point, B – B point, Me – Menton, ZYF - Zygomatic foramen, Co – Condyle, GO – Mandibular gonion, MOR – Medial orbital wall, LOR – Lateral orbital wall, ORF – infra-orbital foramen, GP – Greater palatine foramen, MF – Mental foramen, AR – Anterior ramus, PR – Posterior ramus, R – right, L - left

Mean absolute difference of all measurements was (0.11± 0.12 mm). Bland and Altman Lower limit of agreement ranged from (-0.92 mm to -0.06 mm). Bland and Altman Upper limit of agreement ranged from (0.1 mm to 1.24 mm).

The absolute Dahlberg error between direct linear measurements and linear measurements on stitched CBCT images ranged from (0.07 mm to 0.41 mm). The relative Dahlberg error ranged from (0.2% to 1.8%). Moreover, Intra-class Correlation Coefficient (ICC) ranged from (0.97 to 1.0). ( Table 5).) indicating excellent agreement.

## Discussion

The smaller the scan FOV, the higher the spatial resolution of the image ^8^. Stitching of small CBCT images to create a large image can be very useful to collect the needed cranio-maxillofacial data with small FOV machines ^9^. Increasing the FOV can be done by automatically fusing up to three small FOVs to obtain a larger FOV ^10^.

CBCT ‘‘Stitching’’ option could be useful but whether it is precise enough to obtain accurate and reliable measurements remains doubtful ^11^. Assessing the accuracy of stitched CBCT measurements is infrequently mentioned in current literature, as in the studies conducted by Kopp and Ottl; 2010, Kim *et al.*; 2012, Egbert *et al.*; 2015, and Srimawong *et al.*; 2015 ^5, 8, 10, 12^.

The results of the current study showed that the relative Dahlberg error ranged from 0.2% to a maximum of 1.8%. Consequently, the error was considered small and clinically non-significant as the measurement error in craniofacial imaging is considered clinically acceptable up till the value of 5% ^13, 14^.

The results of the current study go in agreement with the study performed by Srimawong *et al.*; 2015 on 10 dry human mandibles ^12^. Their results showed that, the mean absolute differences between direct measurements and stitched CBCT measurements for vertical and horizontal distances were (0.27±0.24 mm) and (0.34±0.27 mm), respectively. Their results showed that the stitched CBCT measurements were highly accurate comparable to the direct measurements.

In support of the present results, Egbert *et al.*; 2015 ^8^ research revealed that the mean difference between the direct linear measurements and the stitched CBCT measurements was 0.34 mm with a 95% confidence interval of (0.24 to 0.44 mm). They concluded that the precision of stitched CBCT measurements allow accurate construction of implant surgical stents.

Moreover, the results of Kopp and Ottl; 2010 ^10^ further agree with those obtained from the current study. They used an automated method to increase the FOV of CBCT images by stitching three small FOV volumes to obtain a larger FOV one. They concluded that, the stitching software was accurate in the obtained linear measurements.

On the same line of agreement, a study was performed by Kim *et al.*; 2012 ^5^ to investigate whether images of skulls obtained by both manual and automatic stitching of three CBCT images, provided accurate measurements as those obtained by multidetector computed tomography (MDCT). The results showed that the mean difference between automatically stitched CBCT images and the reference images ranged from (-0.8944 mm to -1.0628 mm).

## Conclusion

Stitched CBCT linear measurements were highly comparable to the direct skull measurements. However, a percent of error should be expected from CBCT-derived measurements.

## Data availability

Underlying data is available from Open Science Framework

OSF: Dataset 1. Accuracy of Linear Measurements Obtained from Stitched Cone Beam CT Images Versus Direct Skull Measurements https://doi.org/10.17605/OSF.IO/SUTWK ^15^

## References

[1] Pauwels R Araki K Siewerdsen JH: Technical aspects of dental CBCT: state of the art. *Dentomaxillofac Radiol.* 2015;44(1):20140224. 10.1259/dmfr.20140224 25263643 PMC4277439

[2] Alamri HM Sadrameli M Alshalhoob MA: Applications of CBCT in dental practice: a review of the literature. *Gen Dent.* 2012;60(5):390–400; quiz 401–2. 23032226

[3] Hatcher DC: Operational principles for cone-beam computed tomography. *J Am Dent Assoc.* 2010;141 Suppl 3:3S–6S. 10.14219/jada.archive.2010.0359 20884933

[4] Giesel FL Mehndiratta A Locklin J: Image fusion using CT, MRI and PET for treatment planning, navigation and follow up in percutaneous RFA. *Exp Oncol.* 2009;31(2):106–114. 19550401 PMC2850071

[5] Kim MK Kang SH Lee EH: Accuracy and validity of stitching sectional cone beam computed tomographic images. *J Craniofac Surg.* 2012;23(4):1071–1076. 10.1097/SCS.0b013e31824e2c85 22777443

[6] Brüllmann D Seelge M Schömer E: Alignment of cone beam computed tomography data using intra-oral fiducial markers. *Comput Med Imaging Graph.* 2010;34(7):543–552. 10.1016/j.compmedimag.2010.03.005 20418057

[7] Schropp L Alyass NS Wenzel A: Validity of wax and acrylic as soft-tissue simulation materials used in *in vitro* radiographic studies. *Dentomaxillofac Radiol.* 2012;41(8):686–90. 10.1259/dmfr/33467269 22933536 PMC3528195

[8] Egbert N Cagna DR Ahuja S: Accuracy and reliability of stitched cone-beam computed tomography images. *Imaging Sci Dent.* 2015;45(1):41–7. 10.5624/isd.2015.45.1.41 25793182 PMC4362990

[9] Zaidi H Montandon ML Alavi A: The clinical role of fusion imaging using PET, CT, and MR imaging. *Magn Reson Imaging Clin N Am.* 2010;18(1):133–149. 10.1016/j.mric.2009.09.010 19962098

[10] Kopp S Ottl P: Dimensional stability in composite cone beam computed tomography. *Dentomaxillofac Radiol.* 2010;39(8):512–6. 10.1259/dmfr/28358586 21062945 PMC3520214

[11] Naoumova J Lindman R: A comparison of manual traced images and corresponding scanned radiographs digitally traced. *Eur J Orthod.* 2009;31(3):247–253. 10.1093/ejo/cjn110 19342425

[12] Srimawong P Krisanachinda A Chindasombatjaroen J: Accuracy of linear measurements in stitched versus non-stitched cone beam CT images. *CU Dent J.* 2015;38:93–104. Reference Source

[13] Hilgers ML Scarfe WC Scheetz JP: Accuracy of linear temporomandibular joint measurements with cone beam computed tomography and digital cephalometric radiography. *Am J Orthod Dentofacial Orthop.* 2005;128(6):803–811. 10.1016/j.ajodo.2005.08.034 16360924

[14] Tarazona-Álvarez P Romero-Millán J Peñarrocha-Oltra D: Comparative study of mandibular linear measurements obtained by cone beam computed tomography and digital calipers. *J Clin Exp Dent.* 2014;6(3):e271–4. 10.4317/jced.51426 25136429 PMC4134857

[15] Hamed D Hamdy RM Dessouky SHE: Accuracy of Linear Measurements Obtained from Stitched Cone Beam CT Images Versus Direct Skull Measurements.2019 10.17605/OSF.IO/SUTWK PMC7194353 32399179

