# Supplementary material for: Accuracy of linear measurements obtained from stitched cone beam computed tomography images versus direct skull measurements
Source: F1000Res. 2020 Mar 2;8:166. Originally published 2019 Feb 7. [Version 2] doi: 10.12688/f1000research.17751.2 (PMC7194353; doi:10.12688/f1000research.17751.2)
Supplement: Peer review history

# Peer review: Reviewer response for version 2

**DOI:** 10.5256/f1000research.24950.r60778

**Published:** 2020-04-28

**Authors:** Luca Fiorillo

**Affiliations:** 1 Department of Biomedical and Dental Sciences and Morphological and Functional Imaging, University of Messina, Messina, Italy

Please improve conclusion section with future perspective of this study.

Is the work clearly and accurately presented and does it cite the current literature?

Yes

If applicable, is the statistical analysis and its interpretation appropriate?

Yes

Are all the source data underlying the results available to ensure full reproducibility?

Partly

Is the study design appropriate and is the work technically sound?

Yes

Are the conclusions drawn adequately supported by the results?

Partly

Are sufficient details of methods and analysis provided to allow replication by others?

Yes

Reviewer Expertise:

NA

I confirm that I have read this submission and believe that I have an appropriate level of expertise to confirm that it is of an acceptable scientific standard.

# Peer review: Reviewer response for version 1

**DOI:** 10.5256/f1000research.19409.r55480

**Published:** 2019-11-13

**Authors:** Pathawee Khongkhunthian

**Affiliations:** 1 Center of Excellence for Dental Implantology, Faculty of Dentistry, Chiang Mai University, Chiang Mai, Thailand

The comparative study between direct measurement of the skull and the stitching CBCT data obtained from different view had been well designed and performed. The statistical analysis are appropriate and sufficient to make the conclusion. The study showed the accuracy of stitching method using a commercial software.

 Some of the issues should be raised in the discussion part for more information of the study.

1. The use of dry skull compared to the real clinical situation in term of soft tissue, does the soft tissue impair the clinical accuracy?
2. If comparing the measurements between the stitching technique to the large field of view in CBCT (full skull sensor), how is the accuracy?
3. The limitation of the study should be mentioned.
4. The clinical implication?
5. It should be discussed about the software used, would the results be different if the clinician used the different software?

Is the work clearly and accurately presented and does it cite the current literature?

Yes

If applicable, is the statistical analysis and its interpretation appropriate?

Yes

Are all the source data underlying the results available to ensure full reproducibility?

Yes

Is the study design appropriate and is the work technically sound?

Yes

Are the conclusions drawn adequately supported by the results?

Yes

Are sufficient details of methods and analysis provided to allow replication by others?

Yes

Reviewer Expertise:

dental implantology, biomaterial in tissue regenneration.

I confirm that I have read this submission and believe that I have an appropriate level of expertise to confirm that it is of an acceptable scientific standard.

# Peer review: Reviewer response for version 1

**DOI:** 10.5256/f1000research.19409.r52211

**Published:** 2019-08-12

**Authors:** Luca Fiorillo

**Affiliations:** 1 Department of Biomedical and Dental Sciences and Morphological and Functional Imaging, University of Messina, Messina, Italy

This study is very interesting, and having assessed the reliability of radiographic examinations is very useful for clinical practice.

 We should only understand, as a future perspective, the differences between the different devices used, or between the different exposure settings; it could be mentioned in "Discussion" section as a limitation of the study.

 The work has been well done, and the results are in agreement with other studies in the literature.

- In the "Keywords" section, I recommend using Meshwords (https://meshb.nlm.nih.gov/)
- I suggest to expand the "Introduction" section: Add notes on the history of radiology in maxillofacial and oral medicine ^1^ ^2^. Add clinical notes on the use of three-dimensional radiographic examinations, and how these may influence rehabilitation ^3^. Furthermore it is necessary to subdivide the section into two sub-sections, for example "Background" and "Aim".
- The "Methods" section should be implemented only by improving image quality if possible.
- In the "Conclusions" section, cite a future perspective of the study, and the function of this research in clinical practice.

Is the work clearly and accurately presented and does it cite the current literature?

Yes

If applicable, is the statistical analysis and its interpretation appropriate?

Yes

Are all the source data underlying the results available to ensure full reproducibility?

Partly

Is the study design appropriate and is the work technically sound?

Yes

Are the conclusions drawn adequately supported by the results?

Partly

Are sufficient details of methods and analysis provided to allow replication by others?

Yes

Reviewer Expertise:

NA

I confirm that I have read this submission and believe that I have an appropriate level of expertise to confirm that it is of an acceptable scientific standard, however I have significant reservations, as outlined above.

## References

[1] : Combined surgical reconstruction of the maxilla and mandible for vertical disproportion. *Plast Reconstr Surg*.1970;46(3) : 10.1097/00006534-197009000-00007 252-5 10.1097/00006534-197009000-00007 5448353

[2] : Computers in cephalometric research. *Comput Biol Med*.1970;1(1) :41-9 552458410.1016/0010-4825(70)90015-6

[3] : Dental Restorative Digital Workflow: Digital Smile Design from Aesthetic to Function. *Dent J (Basel)*.2019;7(2) : 10.3390/dj7020030 10.3390/dj7020030 30925698PMC6632039
